# Tracking Cadmium Transfer from Soil to Cup: An Electrochemical Sensing Strategy Based on Bi^3+^-Rich MOFs for Tea Safety Monitoring

**DOI:** 10.3390/foods14213779

**Published:** 2025-11-04

**Authors:** Jiaoling Wang, Zhengyin Ding, Xinxin Wu, Xindong Wang, Hao Li, Minchen Zhu, Xinai Zhang

**Affiliations:** 1 Key Laboratory of Modern Agricultural Equipment, Nanjing Institute of Agricultural Mechanization, Ministry of Agriculture and Rural Development, Nanjing 210014, China; 2 School of Food and Biological Engineering, Jiangsu University, Zhenjiang 212013, China; dzy7143@163.com (Z.D.); 19825549348@163.com (X.W.); w1341501173@163.com (X.W.); 19351703383@163.com (H.L.); zhangxinhuan817@163.com (M.Z.); 3 School of Mechanical Engineering, Jiangsu University, Zhenjiang 212013, China

**Keywords:** cadmium detection, metal–organic framework (MOF), electrochemical sensor, tea safety evaluation, heavy metal migration

## Abstract

Tea is one of the most widely consumed beverages worldwide, yet increasing environmental cadmium (Cd^2+^) contamination poses a serious threat to consumer safety. Understanding the migration pathway of Cd^2+^ from contaminated soils through tea plants into brewed infusions is essential for comprehensive risk assessment across the entire tea supply chain. However, conventional analytical methods for Cd^2+^ detection are often time-consuming, labor-intensive, and unsuitable for rapid or on-site monitoring. In this study, we developed a facile, sensitive, and selective electrochemical sensing platform based on a Bi^3+^-rich metal–organic framework (MOF(Bi)) for reliable Cd^2+^ quantification in various tea-related matrices. The MOF(Bi) was synthesized via a solvothermal method and directly immobilized onto a glassy carbon electrode (GCE) in a one-step modification process. To enhance Cd^2+^ preconcentration, cysteine was introduced as a complexing agent, while Nafion was employed to stabilize the sensing interface and improve reproducibility. The resulting Nafion/cys/MOF(Bi)/GCE sensor exhibited excellent sensitivity with a wide linear range from 0.2 and 25 μg/L, a low detection limit of 0.18 μg/L (*S*/*N* = 3), high selectivity against common interfering ions, and good stability. This platform enabled accurate tracking of Cd^2+^ transfer from polluted garden soil to raw tea leaves and finally into tea infusions, showing strong correlation with ICP-MS results. Our strategy not only offers a practical tool for on-site food safety monitoring but also provides new insights into heavy metal transfer behavior during tea production and consumption.

## 1. Introduction

Tea (*Camellia sinensis*) is one of the most widely consumed non-alcoholic beverages, reaching a global intake of 6800 million kg per year [1]. With a history spanning thousands of years, tea plays a vital role not only in dietary habits but also in cultural traditions across Asia, Africa, the Middle East, and beyond [2,3,4,5,6]. Globally, China is the largest tea producer, with 54% of the tea plantation area and 40% of the tea production [7]. As both the largest producer and consumer of tea, China faces increasing challenges related to tea safety, particularly concerning heavy metal contamination arising from environmental pollution due to rapid industrialization, mining activities, and improper agricultural practices [8,9,10,11,12].

Among various toxic heavy metals, cadmium (Cd) has emerged as a critical contaminant in agricultural products, including tea. Cd is classified as a Group 1 human carcinogen, and chronic exposure even at low levels, can lead to severe health consequences [13,14,15], including renal dysfunction, bone demineralization, oxidative stress, and disruption of essential metal ion homeostasis. Due to its long biological half-life and bioaccumulation potential in edible plant tissues [16,17,18], Cd enters the human body primarily through the food chain [19,20,21,22], posing significant public health risks.

Typical background concentrations of Cd in uncontaminated agricultural soils generally range from 0.01 to 0.5 mg/kg, depending on soil type, geology, and regional environmental conditions [23]. For instance, in natural soils derived from common parent materials, Cd levels are usually below 0.3 mg/kg, with global averages estimated at approximately 0.1–0.3 mg/kg [24,25]. Regarding regulatory limits for Cd in agricultural soils, several countries and regions have established threshold values to protect food safety. For example: in China, the Environmental Quality Standard for Soils (GB 15618-2018 [26]) sets a maximum allowable concentration of 0.3–0.6 mg/kg for Cd in paddy fields and other cultivated lands, depending on soil pH and land use type. The European Union does not currently enforce a universal limit for Cd in soil but recommends precautionary thresholds around 0.8–1.5 mg/kg, depending on land use and soil properties, under its Soil Thematic Strategy [27]. These values highlight the narrow margin between natural background levels and potentially hazardous concentrations, especially in areas affected by industrial emissions, phosphate fertilizer application, or wastewater irrigation factors known to elevate soil Cd content.

To safeguard consumer health, regulatory bodies have established maximum permissible levels of Cd in foodstuffs [28]. Accordingly. the European Union enforces a stricter standard of 0.1 mg/kg in food (EC No 1881/2006 [29]). Similarly, in Brazil, the National Health Surveillance Agency (ANVISA) establishes maximum limits of 20 μg/L for Cd in tea [22]. In China, the National Health Commission specifies that the Cd content in tea should not exceed 0.2 mg/kg (GB 2762-2017, National Food Safety Standard for Contaminants in Foods [30]). However, recent monitoring studies have reported Cd concentrations exceeding these limits in certain tea-growing regions [31], especially those near industrial zones or contaminated soils. To be pointed, available studies indicate that Cd concentrations in tea leaves rarely exceed 1–8 mg/kg dry weight, even when grown in moderately contaminated soils [32]. For brewed tea infusions, due to the low solubility of Cd under typical brewing conditions, only a small fraction (usually <10%) of the total Cd present in dried leaves is leached into the beverage. Consequently, infusion Cd levels are generally much lower typically below 5 μg/L, though higher values have been reported in rare cases using contaminated leaf material [33]. While tea is not a hyperaccumulator [34], its widespread consumption and frequent cultivation in regions with marginal soil quality necessitate careful monitoring of Cd transfer from soil to cup.

Conventional analytical techniques for Cd detection such as inductively coupled plasma mass spectrometry (ICP-MS) and atomic absorption spectroscopy (AAS) [35,36,37,38], offer high accuracy and sensitivity but are limited by their requirement for expensive instrumentation, complex sample preparation, and centralized laboratory settings, making them unsuitable for on-site or real-time monitoring. In contrast, electrochemical methods [39,40,41,42], particularly anodic stripping voltammetry (ASV) [43,44,45,46], have gained growing attention for heavy metal analysis in food matrices due to their portability, low cost, rapid response, and excellent sensitivity. Numerous studies have demonstrated the applicability of ASV for Cd^2+^ detection in rice [47], vegetables [48], milk [49], and herbal infusions [50], offering a promising alternative to traditional methods.

Despite these advances, ASV approaches often rely on mercury-based electrodes [51], which, although effective, pose environmental and health risks. Recent efforts have shifted toward the use of bismuth-based materials [52], which are less toxic and offer comparable sensitivity. However, the synthesis and modification of these materials often involve complex procedures, limiting their practical application in field settings or routine monitoring. To address these limitations, metal–organic frameworks (MOFs) have recently emerged as ideal platforms for electrochemical sensing owing to their high surface area, tunable porosity, and abundant active sites [8,53,54,55], which enable efficient preconcentration and electron transfer during voltammetric detection. In particular, MOF-based sensors have demonstrated significant advantages in the determination of heavy metal ions [56,57]. The porous architecture of MOFs allows for size- and shape-selective adsorption of target analytes, while functional groups within the framework can coordinate specifically with metal ions, enhancing both sensitivity and selectivity. Moreover, when integrated into electrode interfaces, MOFs facilitate the accumulation of metal ions on the sensor surface through ion-exchange or complexation mechanisms, thereby amplifying the electrochemical signal during ASV measurement.

Inspired by the advantages of MOFs in sensing applications, this work developed a facile, sensitive, and selective electrochemical sensor based on a Bi^3+^-rich MOF (MOF(Bi)) for reliable quantification of Cd^2+^ in tea-related matrices. The MOF(Bi) was synthesized via a solvothermal method and directly immobilized onto a glassy carbon electrode (GCE) in a one-step modification process. Notably, Bi^3+^-rich MOFs combine the advantages of intrinsic bismuth sources with structural stability, enabling simplified sensor fabrication without the need for additional Bi^3+^ electrolyte addition during measurement. Cysteine (cys) was introduced as a Cd^2+^-selective chelating agent to enhance preconcentration, while Nafion was used to improve membrane stability and anti-fouling properties. The resulting sensor was systematically evaluated for its performance in detecting Cd^2+^ across the entire tea supply chain from contaminated garden soil, raw tea leaves, to brewed tea infusions. The proposed approach provides a promising tool for evaluating Cd^2+^ contamination across the tea production and consumption chain, offering a reliable and scalable solution for food safety monitoring.

## 2. Materials and Methods

### 2.1. Reagents and Materials

Cysteine (cys), bismuth nitrate pentahydrate (Bi(NO_3_)_3_·5H_2_O), 1,3,5-benzenetricarboxylic acid (H_3_BTC), sodium acetate, anhydrous ethanol, and other reagents were purchased from Sinopharm Chemical Reagent Co., Ltd. (Shanghai, China). A 5% Nafion solution was procured from Sigma-Aldrich. All the reagents used were of analytical reagent grade and all solutions were prepared with pure water from Millipore (Milli-Q, 18.2 MΩ cm).

### 2.2. Apparatus

The scanning electron microscope (SEM, FEI, Hillsboro, OR, USA) used for morphological analysis was a Quanta 250 FEG model (FEI). X-ray photoelectron spectroscopy (XPS) measurements were conducted using an AXIS-Ultra DLD instrument (Shimadzu, Kratos, Kyoto, Japan). Electrochemical experiments were performed with a CHI 630D electrochemical workstation consisting of the modified glassy carbon electrode (GCE, *Φ* = 3 mm) as the working electrode, a saturated calomel electrode (SCE) as the reference, and a platinum wire as the auxiliary (Chenhua Instrument Co., Ltd., Shanghai, China). Magnetic stirring was achieved using an HJ-2 magnetic stirrer (Zhongda Instrument Factory, Jiangsu Zhongda Instrument Technology Co., Ltd., Changzhou, China), while vacuum drying was performed in a DZF-6021 vacuum oven (Shanghai Kuntian Laboratory Instrument Co., Ltd., Shanghai, China). A high-speed centrifuge (H1850, Xiangyi Centrifuge Instrument Co., Ltd., Changsha, China) and a vortex mixer (XH-C, Jintan Medical Instrument, Changzhou Jintan Liangyou Instrument Co., Ltd., Changzhou, China) were employed for sample preparation.

### 2.3. Preparation of MOF(Bi)

First, 0.2213 g of Bi(NO_3_)_3_·5H_2_O and 0.1550 g of H_3_BTC were accurately weighed and transferred into a polytetrafluoroethylene (PTFE) liner of a high-pressure reactor [53,55,58]. Subsequently, 15 mL of anhydrous ethanol was added to dissolve the reagents, and the mixture was allowed to stand undisturbed at room temperature for 10 h. The reaction was then carried out in an oil bath at 120 °C for 12 h. After the reaction, the product was centrifuged three times using anhydrous ethanol, with the centrifuge set at a speed of 6000 rpm for 5 min per cycle. The resulting white powder sample of MOF(Bi) was dried at 60 °C in a vacuum oven for 2 h. For further use, 5 mg of the synthesized MOF(Bi) was accurately weighed and added to a solution containing anhydrous ethanol and ultrapure water in a volume ratio of 6:4 (5 mL total). The mixture was sonicated to achieve uniform dispersion, yielding an MOF(Bi) suspension. This suspension was stored at 4 °C in a refrigerator for subsequent experiments.

### 2.4. Preparation of Sensing Interface

Prior to modifying glassy carbon electrode (GCE), it was subjected to a polishing treatment [41,54]. The electrode was first polished to a mirror-like finish using Al_2_O_3_ powder with particle sizes of 1 μm and 0.05 μm. Subsequently, the electrode surface was ultrasonically cleaned for 90 s in a sequence of ultrapure water, diluted nitric acid (1:1 *v*:*v*), and ultrapure water [59,60]. Following this, the electrode was cycled 80 times in a 0.50 M diluted sulfuric acid solution at a scan rate of 100 mV/s within the potential range of −0.2 to 1.6 V to remove any impurities that might be present on the electrode surface.

The modification process of the electrode surface was carried out as follows [44,61,62]: Initially, 5 μL of a MOF(Bi) suspension was pipetted onto the surface of GCE and dried slowly under an infrared drying lamp. After the surface had dried, 3 μL of a 0.01 M cysteine solution (cys) was accurately pipetted onto the electrode surface and dried similarly under the infrared lamp. Finally, 2 μL of a 0.16% Nafion solution was pipetted onto the electrode surface and allowed to dry naturally at room temperature, resulting in the fabrication of a Nafion/cys/MOF(Bi)/GCE sensor.

### 2.5. Electrochemical Measurement

All electrochemical measurements were conducted using a CHI 630D electrochemical workstation, employing a conventional three-electrode system for detection. A saturated calomel electrode (SCE) served as the reference electrode, a platinum wire electrode as the auxiliary electrode, and a glassy carbon electrode (GCE) as the working electrode [63,64,65]. A 0.1 M acetic acid–sodium acetate (HAc-NaAc) buffer solution at pH 4.5 was used as the electrolyte. Initially, Cd^2+^ ions in the solution were pre-concentrated via potentiostatic deposition with the following parameters: a constant potential of −1.4 V and a deposition time of 600 s. The potentiostatic curve data were recorded, and the resulting current-time (i-t) curves were integrated using Origin data analysis software (Origin 2025) to determine the transferred charge quantity (Q). Subsequently, the determination of the pre-concentrated Cd was performed with the following parameters: a scan range from −1.0 V to −0.4 V, a pulse frequency of 15 Hz, and a pulse amplitude of 25 mV.

### 2.6. Preparation of Samples

(1) Preparation of Cadmium-Contaminated Soil [66,67,68,69]: To simulate realistic environmental contamination scenarios, cadmium-spiked soil samples were prepared under controlled conditions for pot experiments. A loamy garden soil was collected from a non-contaminated agricultural site, air-dried at room temperature, and manually homogenized after removal of stones, roots, and other debris. The soil was then sieved through a 2 mm mesh to ensure uniformity. To establish different levels of Cd contamination, analytical-grade cadmium nitrate tetrahydrate (Cd(NO_3_)_2_·4H_2_O) was dissolved in deionized water and uniformly sprayed onto the soil at target concentrations of 10 mg/kg (C10), 15 mg/kg (C15), and 20 mg/kg (C20) on a dry weight basis. After spiking, the soils were thoroughly mixed, aged for four weeks under controlled moisture conditions (60% field capacity), and stored for equilibration before use. This aging period allowed for better metal-soil interaction and minimized acute toxicity effects during plant growth.

(2) Cultivation of Tea in Cadmium-Contaminated Soil: Healthy tea cuttings were transplanted into pots containing Cd-spiked soils (C10, C15, C20) with five replicates per treatment. Plants were grown under controlled greenhouse conditions for 24 months to simulate long-term Cd exposure. Growth parameters included a 12/12 h light/dark cycle, 60–70% relative humidity, and temperature at 25 ± 2 °C. Natural light was supplemented with full-spectrum LED lights to maintain PAR > 400 μmol/m^2^/s. Plants were irrigated with deionized water; no fertilizers or pesticides were applied to avoid interference with Cd uptake. Monthly monitoring included growth (height, leaf number) and phytotoxicity symptoms (e.g., chlorosis, necrosis). After 24 months, the first harvest was conducted by plucking the apical bud and two youngest leaves, followed by subsequent harvests every three months.

(3) Harvesting and Drying of Tea Leaves: At harvest, fresh tea leaves (including the apical bud and two youngest leaves) were collected from each plant, immediately placed on ice, and transported to the laboratory for processing. Subsamples were reserved for fresh weight analysis and moisture content determination. The remaining leaves were subjected to standard withering and drying procedures to simulate commercial tea production. Briefly, the leaves were spread evenly on trays and withered at room temperature (25 °C) for 18 h to reduce moisture content. They were then fixed by steaming for 3 min followed by drying in an oven at 105 °C until constant weight was achieved. The dried tea samples were ground into a fine powder using a ceramic mill, passed through a 100-mesh sieve, and stored in sealed polyethylene bags at 4 °C pending further analysis. This process ensured sample homogeneity and stability while mimicking real-world tea manufacturing protocols.

(4) Mineralization of Samples [70,71,72,73]: Prior to electrochemical and instrumental analysis, all solid samples including contaminated soils and raw/dried tea leaves, were subjected to microwave-assisted acid digestion to extract total Cd content. Approximately 1.0 g of each sample was accurately weighed into a polytetrafluoroethylene (PTFE) high-pressure digestion vessel. Then, 5.0 mL of concentrated nitric acid (HNO_3_, ≥69%) was added, and the mixture was pre-digested overnight at room temperature. The vessels were sealed and placed in a microwave digestion system (MARS 6, CEM) following the program: ramped to 180 °C over 20 min, held at 180 °C for 20 min, then cooled passively. After cooling, the digests were transferred to clean centrifuge tubes, diluted to 50 mL with 0.1 M acetic acid–sodium acetate buffer (pH 4.5), and filtered through a 0.22 μm nylon membrane. The resulting solutions were stored at 4 °C and analyzed within 24 h using the developed electrochemical sensor.

(5) Preparation of Infusions and Related Extracts [74,75,76]: To evaluate Cd migration into the final beverage, tea infusions were prepared following a standardized brewing protocol that simulates typical consumer practices. In brief, 3.0 g of powdered dried tea leaves from each treatment group were placed into separate 200 mL conical flasks, and 150 mL of freshly boiled ultrapure water (95–100 °C) was poured over the leaves. The mixtures were allowed to steep for 30 min under gentle stirring to ensure consistent extraction. After brewing, the infusions were immediately filtered through a 0.22 μm microporous membrane filter, cooled to room temperature, and stored in acid-washed polypropylene vials prior to analysis. No pH adjustment or preservatives were used to maintain natural infusion conditions. The obtained tea extracts were directly analyzed using the electrochemical sensor for Cd^2+^ quantification.

For comparative purposes, the mineralized samples (soil, tea leaves, and infusions) were analyzed for total cadmium content using inductively coupled plasma mass spectrometry (ICP-MS, Thermo Scientific iCAP RQ, Waltham, MA, USA) under the following operating conditions: RF power: 1550 W, plasma gas flow rate: 15 L/min (argon), auxiliary gas flow rate: 0.8 L/min, nebulizer gas flow rate: 1.05 L/min, spray chamber temperature: 2 °C, sample uptake rate: 0.4 mL/min, dwell time: 100 ms per isotope. All ICP-MS data were used solely for cross-validation of the electrochemical sensor results.

## 3. Results and Discussion

### 3.1. Principle of Electrochemical Sensing Toward Cd^2+^ Testing

**Scheme 1:**
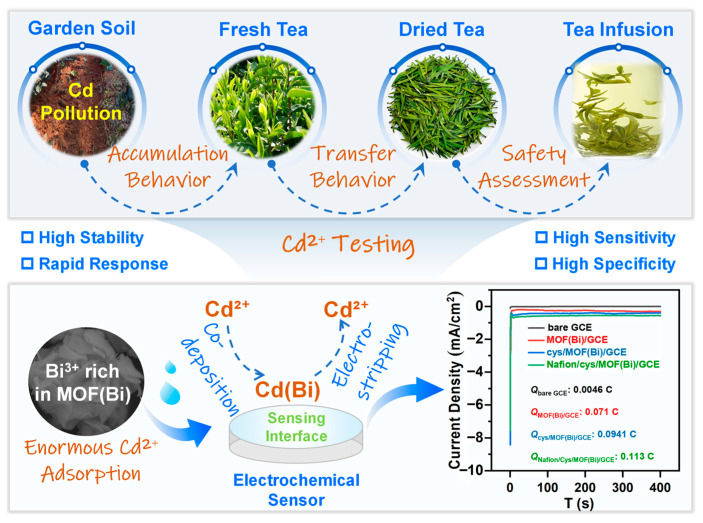
Schematic diagram for Cd^2+^ testing in tea using MOF(Bi)-based sensing interface.

The schematic diagram for the assessment of Cd^2+^ using a MOF(Bi)-modified electrode is illustrated in Scheme 1. Initially, the MOF(Bi) material is coated onto the electrode surface. Compared to the complex preparation process of conventional bismuth film electrodes, the method employed in this experiment is straightforward, achieving bismuth film modification in a single step. Moreover, MOF(Bi) exhibits numerous active sites that facilitate Cd^2+^ adsorption and demonstrate insensitivity to dissolved oxygen, thereby enhancing the sensitivity of Cd^2+^ detection. Subsequently, cysteine (cys) is coated onto the electrode surface. Given that cysteine contains abundant amino, carboxyl, and hydroxyl groups, it can specifically bind to Cd^2+^, thus promoting the enrichment of Cd^2+^ on the glassy carbon electrode (GCE) surface. Finally, due to the film-forming properties of Nafion, MOF(Bi) is firmly anchored onto the electrode surface. Leveraging the strong adsorption capacity of the Nafion-, MOF(Bi)-, and cys-modified electrode toward Cd^2+^, the stripping current signal is amplified, enabling sensitive detection of Cd^2+^ for disclosing accumulation behavior from garden soil to fresh tea, transfer behavior during tea processing, and safety assessment in tea infusion.

### 3.2. Characterization of MOF(Bi)

**Figure 1:**
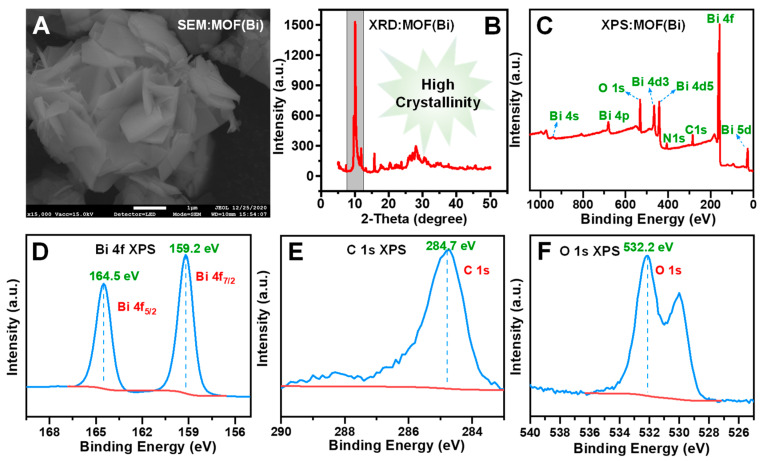
Characterization of MOF(Bi). (**A**) SEM image, (**B**) XRD, (**C**) XPS full-spectra, (**D**) high-resolution Bi 4f XPS spectra, (**E**) C 1s XPS spectra, (**F**) O 1s XPS spectra.

The morphology and elemental composition of MOF(Bi) were characterized using scanning electron microscopy (SEM), X-ray diffraction (XRD), and X-ray photoelectron spectroscopy (XPS). Figure 1A depicts the SEM images of MOF(Bi). As observed in the images, the material consists of numerous irregularly stacked lamellar structures with a clean, impurity-free background. The MOF(Bi) exhibits a nanosheet distribution, with thicknesses ranging from 0 to 150 nm and a smooth surface. Figure 1B depicts the XRD pattern of MOF(Bi), where sharp and intense diffraction peaks are evident, indicating the good crystallinity of MOF(Bi). Figure 1C illustrates the elemental composition analysis of MOF(Bi) via XPS measurement. The characteristic peaks at 159.2 eV and 164.5 eV correspond to Bi 4f_7_/_2_ and Bi 4f_5_/_2_, respectively (Figure 1D), originating from Bi^3+^ in MOF(Bi). The peak at 284.7 eV is attributed to amorphous carbon (Figure 1E), while the peak at 532.2 eV is associated with oxygen in the Bi-O bond (Figure 1F). The XPS data indicate that the material is predominantly composed of bismuth [77], providing a substantial source of bismuth for subsequent electrochemical assay. The presence of Bi^3+^ supports the mechanism of bismuth-film-assisted Cd^2+^ co-deposition, while the nanosheet morphology enhances surface area and mass transfer.

### 3.3. Electrochemical Properties of MOF(Bi)-Anchored Interface

**Figure 2:**
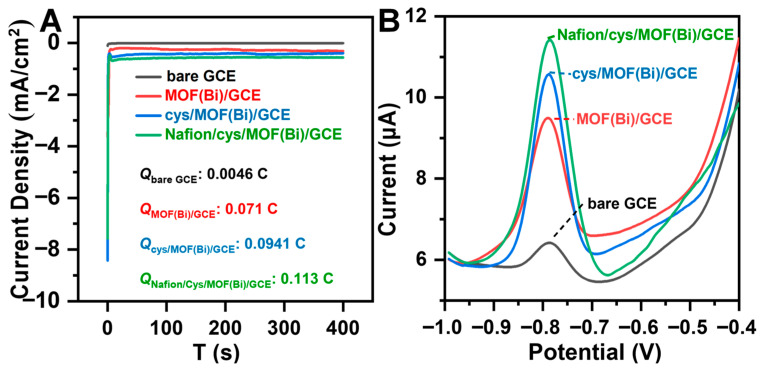
(**A**) Potentiostatic curve of Cd^2+^ deposition, (**B**) SWV curves for 60 μg/L Cd^2+^ assay from bare GCE, MOF(Bi)/GCE, cys/MOF(Bi)/GCE, Nafion/cys/MOF(Bi)/GCE, respectively.

The detection performance of various modified electrodes was characterized using potentiostatic measurement and square-wave voltammetry (SWV). As illustrated in Figure 2A, the charge transfer quantity was determined by integrating the current-time (i-t) curves of different modified electrodes. The calculated charge transfer quantities were as follows: Q_(bare GCE)_ = 0.0046 C, Q_(MOF(Bi)/GCE)_ = 0.071 C, Q_(cys/MOF(Bi)/GCE)_ = 0.094 C, and Q_(Nafion/cys/MOF(Bi)/GCE)_ = 0.113 C. The amount of Cd^2+^ reduced on the electrode surface was calculated using Faraday’s law:

Q = nN_a_eZ

In the equation, Q represents the transferred charge quantity, n denotes the amount of electrodeposited substance, N_a_e is Faraday’s constant, and Z stands for the charge number of a single particle. The calculated amounts of Cd^2+^ deposited were as follows: n_(bare GCE)_ = 0.023 μmol, n_(MOF(Bi)/GCE)_ = 0.368 μmol, n_(cys/MOF(Bi)/GCE)_ = 0.487 μmol, and n_(Nafion/cys/MOF(Bi)/GCE)_ = 0.585 μmol. It was observed that n_(Nafion/cys/MOF(Bi)/GCE)_ > n_(cys/MOF(Bi)/GCE)_ > n_(MOF(Bi)/GCE)_ > n_(bare GCE)_, indicating that the Nafion/cys/MOF(Bi)-modified sensing interface enhances the deposition of Cd^2+^ from the solution.

Figure 2B presents the SWV plots from different modified electrodes, where the stripping potential of Cd^2+^ is observed around −0.8 V. As the electrode surface was progressively modified, the response current increased continuously, with the Nafion/cys/MOF(Bi)-modified electrode exhibiting the highest stripping current. This could be attributed to two main factors: firstly, the bismuth ions in MOF(Bi) can co-deposit with Cd^2+^ from the solution onto the electrode surface, resulting in a greater deposition of Cd^2+^ compared to the bare electrode; secondly, cysteine can undergo a complexation reaction with Cd^2+^ in the solution, facilitating the enrichment of more Cd^2+^ on the electrode surface. Additionally, the excellent film-forming properties of Nafion firmly anchor MOF(Bi) onto the electrode surface, significantly increasing the specific surface area of the electrode and leading to a substantial increase in the Cd^2+^ current, thereby enhancing the sensor’s sensitivity for the Cd^2+^ assay.

### 3.4. Optimization of Experimental Conditions

**Figure 3:**
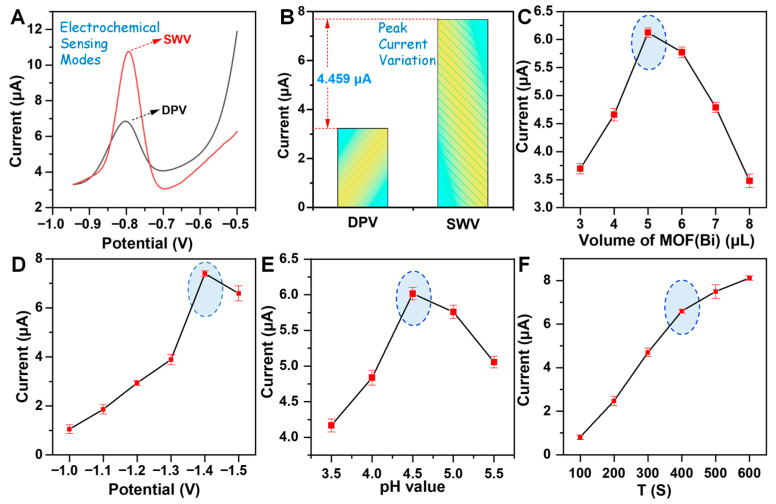
(**A**) Electrochemical sensing modes for stripping currents, (**B**) the corresponding peak current variation between DPV and SWV. Effects of (**C**) volume of MOF(Bi), (**D**) deposition voltage, (**E**) pH value, (**F**) deposition time on 60 μg/L Cd^2+^ assay.

To achieve optimal analytical sensitivity for Cd^2+^, a comparative study was conducted using differential pulse voltammetry (DPV) and SWV for the determination of enriched Cd. As illustrated in Figure 3A,B, under the same conditions including Cd^2+^ concentration, electrode modification, and potentiostatic deposition, the stripping current obtained using the SWV pattern was significantly higher than that of the DPV mode. This indicated that the SWV pattern exhibited superior sensitivity. Consequently, SWV was employed as the electrochemical sensing modes for generating stripping currents associated with Cd^2+^ in the subsequent experiments.

The quantity of MOF(Bi) modified on the sensing interface is a crucial factor influencing the deposition of Cd^2+^. As depicted in Figure 3C, SWV response signals were investigated by modifying the electrode surface with 3 μL, 4 μL, 5 μL, 6 μL, 7 μL, and 8 μL of MOF(Bi) (1 mg/mL), respectively. It can be observed that the current exhibited an increasing trend within the range of 3 to 5 μL, reaching a maximum at 5 μL. This was attributed to the formation of an “alloy” between Bi^3+^ in MOF(Bi) and Cd^2+^ in the solution, which enhanced the deposition of Cd^2+^ on the electrode surface. Within the range of 5 to 8 μL, the current gradually decreased, presumably due to the increased amount of MOF(Bi) impeding electron transfer. Consequently, 5 μL of MOF(Bi) was selected as the optimal quantity for modifying the electrode.

Furthermore, the deposition potential and deposition time were optimized. As illustrated in Figure 3D, the current gradually increased as the voltage increased from −1.0 V to −1.4 V. At excessively low potentials, the applied potential difference was insufficient to drive effective reduction, resulting in reduced deposition of Cd^2+^. When the voltage exceeded −1.4 V, the current decreased. This decrease was attributed to the formation of numerous bubbles on the working electrode surface due to hydrogen evolution, which enveloped the electrode. These bubbles reduced the number of active sites on the electrode surface and hindered the deposition of Cd^2+^, leading to a decrease in current. Therefore, −1.4 V was chosen as the optimal deposition voltage.

Additionally, the pH value of the buffer solution significantly influences the sensor’s assay performance. The variation in the SWV response current with respect to pH was investigated, as depicted in Figure 3E. It was observed that the current gradually increased within the pH range from 3.5 to 4.5, reaching a maximum at pH 4.5. As the pH continued to increase beyond 4.5, the current began to decrease due to the hydrolysis of metal ions. Consequently, pH 4.5 was selected as the optimal pH for the experiment.

Finally, Figure 3F presents the optimization of the deposition time, where it can be observed that the SWV response signal gradually increased within the range of 100 s to 600 s. The current increase trend slowed down beyond 400 s, prompting the selection of 400 s as the optimal deposition time.

### 3.5. Electrochemical Sensing Toward Cd^2+^

**Figure 4:**
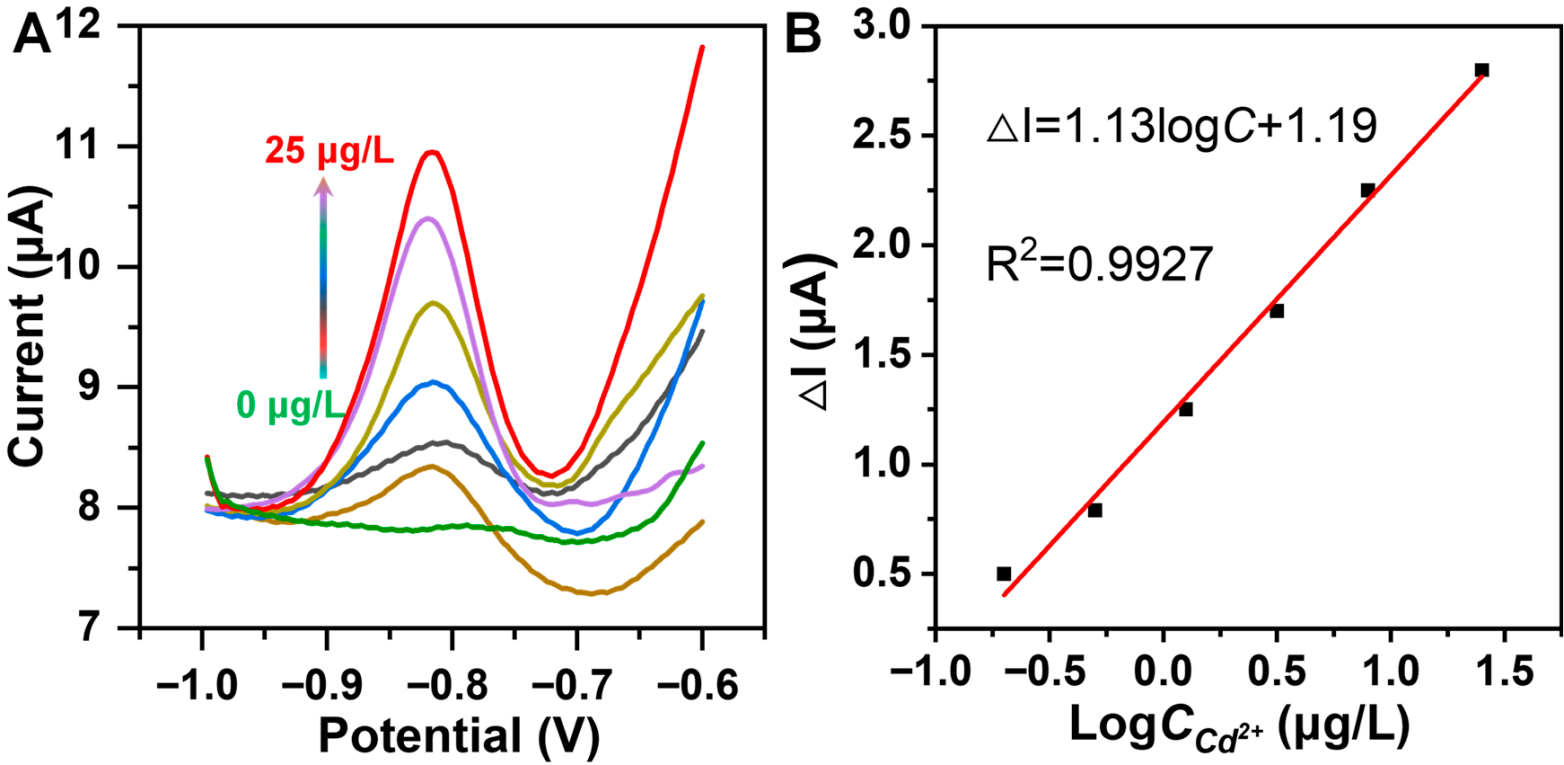
(**A**) SWV response for detecting Cd^2+^ at different concentrations, (**B**) the corresponding standard curve.

Under optimal experimental conditions, the proposed pattern was applied to the detection of Cd^2+^ at various concentrations. As illustrated in Figure 4A, with increasing Cd^2+^ concentrations, the SWV response current enhanced obviously. A good linear relationship was observed between the logarithm of the Cd^2+^ concentration and the net peak current (ΔI), which corresponds to the difference between the anodic stripping peak current in the presence of Cd^2+^ and the blank signal (Figure 4B). The linear regression equation for Cd^2+^ analysis was determined to be ΔI = 1.13 logC_Cd_^2+^ + 1.19 (R^2^ = 0.9927). The detection limit of the proposed technique was calculated to be 0.18 μg/L based on the signal-to-noise ratio (*S*/*N* = 3), and the linear range was between 0.2 and 25 μg/L. Compared to methods reported in the literature [78,79,80,81,82], the MOF(Bi)-based electrochemical analysis method exhibited obvious merits including high sensitivity and rapid response, making it suitable for Cd^2+^ assay.

### 3.6. Stability, Reproducibility, Specificity

**Figure 5:**
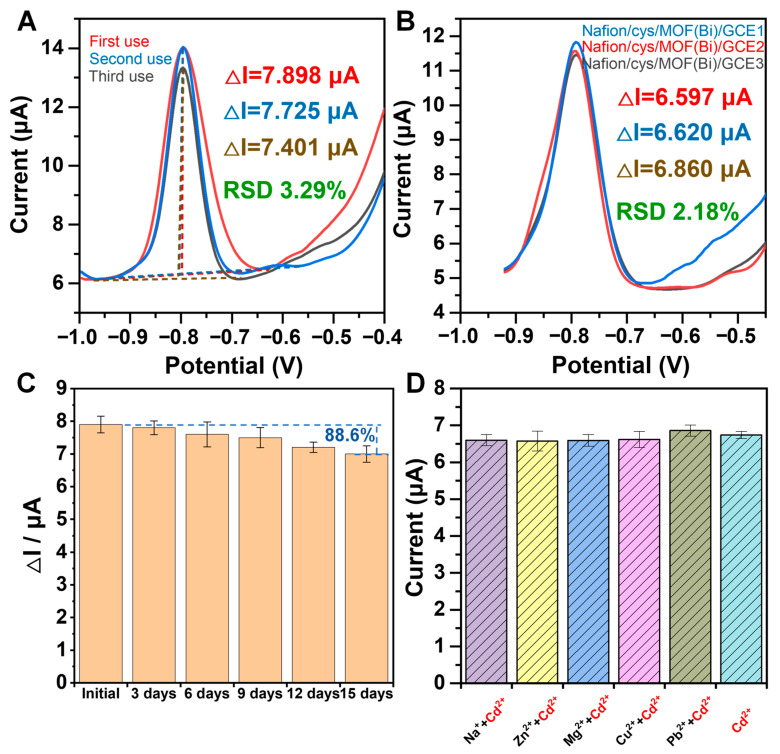
(**A**) Stability, (**B**) reproducibility, (**C**) lifetime testing, (**D**) specificity of the proposed sensor toward Cd^2+^ testing.

The stability of the sensing interface was investigated by employing the prepared pattern for Cd^2+^ assay and reusing for three times, as illustrated in Figure 5A. The relative standard deviation (RSD) of the peak current was calculated to be 3.29%. These results indicate that the sensor possesses good stability.

Additionally, three Nafion/cys/MOF(Bi)-modified electrodes were used for Cd^2+^ assay, respectively. As shown in Figure 5B, the RSD of the peak current was calculated to be 2.18%, verifying good reproducibility of the sensor. When stored at 4 °C, the electrode retains over 88% of its original response after 15 days (Figure 5C), demonstrating acceptable shelf life for routine use due to the robust properties of the nanostructures. To evaluate the sensor’s specificity for Cd^2+^ assay, control experiments were conducted by introducing interfering metal ions. Specifically, Na^+^, Zn^2+^, Mg^2+^, Cu^2+^, and Pb^2+^ (300 μg/L) were added to Cd^2+^ solution. As depicted in Figure 5D, there was little difference in the SWV response current between the solution with interfering ions and that without interfering ions. Consequently, these results confirmed that the prepared sensor in this experiment exhibited good specificity for Cd^2+^ monitoring.

### 3.7. Detection of Cd^2+^ in Real Samples

To investigate the application potential of the proposed sensor [83], it was utilized for Cd^2+^ assay in garden soil, fresh tea, dried tea, and tea infusion, respectively. The experimental results are presented in Table 1. As the concentration of cadmium in the soil increased, the accumulation of cadmium in fresh tea exhibited a gradual upward trend. After tea processing, the slightly elevated cadmium content in dried tea may result from the higher water content in fresh tea leaves, which decreased substantially after the drying process. Following tea brewing, only a minor fraction of cadmium was transferred into the tea infusion, representing less than 7.5% of the total cadmium content in dried tea. The RSD of the tests is no more than 5.6%, indicating that the proposed sensor has acceptable reproducibility for Cd^2+^ assay. The relative error (RE) is less than 8.6%, verifying the good accuracy of the proposed strategy.

It should be noted that the present study utilized a single soil type spiked with known concentrations of Cd^2+^ for proof-of-concept validation. While this allowed us to control variables and assess Cd migration under standardized conditions, the findings may not directly translate to other soil types with differing physicochemical properties (e.g., pH, clay content, organic matter). Future work will involve testing the sensor in multiple soil types representative of major tea-producing regions worldwide.

## 4. Conclusions

In conclusion, we have developed a facile, sensitive, and selective electrochemical sensor based on a Bi^3+^-rich MOF combined with cysteine and Nafion for the reliable quantification of Cd^2+^ in tea-related matrices. The one-step electrode modification strategy eliminates the need for external Bi^3+^ addition, offering a simplified and eco-friendly alternative to traditional bismuth-film or mercury-based electrodes. The integration of cysteine enables selective Cd^2+^ preconcentration via complexation, while Nafion enhances membrane stability and anti-fouling properties, that critical for long-term and reproducible sensing performance. The sensor was successfully applied to monitor cadmium migration from contaminated garden soil through raw tea leaves to brewed infusions, showing strong agreement with ICP-MS results. This capability provides valuable insight into heavy metal transfer behavior during tea production and consumption. Given its portability, low cost, and high accuracy, we recommend this platform as a promising tool for food safety evaluation, particularly in scenarios requiring rapid and decentralized tracking cadmium transfer from soil to cup. Moreover, given its high sensitivity and selectivity, this sensing strategy shows great potential for Cd^2+^ detection in diverse environmental, agricultural, and food safety applications beyond tea, such as water resources, edible crops, and plant-derived products.

## Figures and Tables

**Table 1:** Electrochemical sensing toward Cd^2+^ testing from soil and tea samples (*n* = 3).

Soils	Measured Value in Soil (mg/kg)				Measured Value in Fresh Tea (mg/kg)				Measured Value in Dried Tea (mg/kg)				Measured Value in Tea Infusion (mg/kg)			
	ICP-MS	Sensor	RSD (%)	RE (%)	ICP-MS	Sensor	RSD (%)	RE (%)	ICP-MS	Sensor	RSD (%)	RE (%)	ICP-MS	Sensor	RSD (%)	RE (%)
C_10_	11.07	10.18	4.6	8.04	2.72	2.58	5.0	5.15	4.62	4.39	5.1	4.98	0.35	0.32	4.7	8.57
C_15_	14.4	15.12	5.6	5.00	3.29	3.45	4.8	4.86	4.98	4.62	5.4	7.23	0.37	0.34	4.9	8.11
C_20_	22.8	21.56	5.2	5.44	3.57	3.49	5.3	2.24	6.23	6.47	4.5	3.85	0.41	0.39	5.5	4.88

## Data Availability

The original contributions presented in this study are included in the article. Further inquiries can be directed to the corresponding authors.
